# Antifungal activity of essential oils from Iranian plants against fluconazole-resistant and fluconazole-susceptible *Candida albicans*

**Published:** 2016

**Authors:** Aghil Sharifzadeh, Hojjatollah Shokri

**Affiliations:** 1 *Mycology Research Center, Faculty of Veterinary Medicine, University of Tehran, Tehran, Iran*; 2 *Faculty of Veterinary Medicine, Amol University of Special Modern Technologies, Amol, Iran*

**Keywords:** *Oral candidiasis*, *Candida albicans*, *Essential oils*, *Origanum vulgare*, *Fluconazole-resistant isolates*

## Abstract

**Objective:**

The purpose of this study was to assay the antifungal activity of selected essential oils obtained from plants against both fluconazole (FLU)-resistant and FLU-susceptible *C. albicans* strains isolated from HIV positive patients with oropharyngeal candidiasis (OPC).

**Materials and Methods:**

The essential oils were obtained by hydrodistillation method from *Myrtus communis* (*My. communis*), *Zingiber officinale roscoe* (*Z. officinale roscoe*), *Matricaria* *chamomilla* (*Ma. chamomilla*), *Trachyspermum ammi* (*T. ammi*) and *Origanum vulgare* (*O. vulgare*). The susceptibility test was based on the M27-A2 methodology. The chemical compositions of the essential oils were obtained by gas chromatography- mass spectroscopy (GC-MS).

**Results:**

In GC-MS analysis, thymol (63.40%), linalool (42%), α-pinene (27.87%), α-pinene (22.10%), and zingiberene (31.79%) were found to be the major components of *T. ammi*, *O. vulgare*, *My. communis*, *Ma. chamomilla* and *Z. officinale roscoe*, respectively. The results showed that essential oils have different levels of antifungal activity. *O. vulgare* and *T. ammi* essential oils were found to be the most efficient (P<0.05). The main finding was that the susceptibilities of FLU-resistant *C. albicans* to essential oils were higher than those of the FLU-susceptible yeasts.

**Conclusion:**

Results of this study indicated that the oils from medicinal plants could be used as potential anti FLU-resistant C. albicans agents.

## Introduction

Oropharyngeal candidiasis (also known as oral thrush or OPC) is a common opportunistic mycosis of *Candida* species on, the mucous membranes of the mouth (William and Timothy, 2006 ▶). *Candida* *albicans* (*C. albicans*) is the most common species of yeast isolated from patients with oral candidiasis. The incidence of opportunistic infections due to *C. albicans* and other *Candida* species has been increasing (Fidel, 2006 ▶). Oral candidiasis is the most common HIV-related oral lesion and most patients are infected with a strain originally present as a commensal of the oral cavity (Wabe et al., 2011 ▶). The spectrum of *Candida* infection is diverse, starting from asymptomatic colonization to pathogenic forms. The first step in the development of a *Candida* infection is colonization of the mucocutaneous surfaces. HIV infection is not only associated with increased colonization rates but also with the development of overt disease (Khan et al., 2012 ▶). During the course of HIV infection, the rate of *Candida* infection is inversely related to the CD4 counts of the patient which in turn depends on the use of anti-retroviral treatment. HIV-positive patients carry more and a greater variety of yeasts than HIV-negative subjects (Pappas et al., 2003 ▶). Early recognition and prophylactic treatment may prevent serious morbidity and fatal consequences in high risk patients. Polyene antibiotics have been the initial choice of antifungals for half a century. Recently, the azoles have been used to treat patients with systemic fungal infection. Fluconazole (FLU) is considered well tolerated and showed to be effective in the treatment of OPC (Koletar et al., 1990 ▶). The prolonged management of oral candidiasis in HIV patients might cause the development of drug resistance candidiasis (Hamza et al., 2008 ▶). 

Essential oil is referred to any concentrated, hydrophobic (immiscible with water), typically lipophilic (oil or fat soluble) liquid of plants that contains highly volatile aroma compounds and carries a distinctive scent, flavor, or essence of the plant. This large and diverse class of oils is also referred to as volatile oils or ethereal oils. Essential oils are found in diverse parts of plants including leaves, seeds, flowers, roots and barks. For the plant, essential oils are thought to be vital for the life of the plant, containing compounds that help to fight parasites and infections containing anti-bacterial, anti-fungal, and anti-parasitic properties. For people, essential oils are used in perfumes, cosmetics, and bath products, for flavoring food and drink, for scenting incense and household cleaning products and for medicinal purposes. Interest in essential oils has revived in recent decades, with the popularity of aromatherapy, a branch of alternative medicine which claims that the specific aromas carried by essential oils have curative effects (Abdurahman et al., 2013 ▶).

Antimicrobial properties have been reported more frequently in a wide range of plant extracts and essential oils and natural products in an attempt to discover new chemical classes of antifungal drugs that could resolve these problems (Ali et al., 2001 ▶). Essential oils are very complex natural mixtures, which can contain about 20-60 components at quite different concentrations. They are characterized by two or three major components at fairly high concentrations (20-70%) when compared with other components present in traces (Bakkali and Averbeck, 2008 ▶). According to the literature, the investigation of natural products activity against *Candida* species increased significantly in the last 10 years, with the investigation of approximately 258 plant species from 94 families (Feldmesser, 2003 ▶; Duarte et al., 2008 ▶). Plants from Iranian biomes have also been used as natural medicines by local populations in the treatment of several diseases (Jafari et al., 2003 ▶; Khosravi et al., 2008 ▶). However, despite the rich flora, the antifungal activity of these plants has not been extensively studied. The screening programs for some under-represented targets, such as antifungal activity, may yield candidate compounds for developing new antimicrobial drugs. To explore the anti-*Candida* plants, this study was conducted to screen the antifungal activity of five plant essential oils including *Myrtus communis* (*My. communis*), *Zingiber officinale roscoe* (*Z. officinale* *roscoe*), *Matricaria chamomilla* (*Ma. chamomilla*), *Trachyspermum ammi* (*T. ammi*) and *Origanum vulgare* (*O. vulgare*) against *C. albicans* isolated from oral cavity of HIV positive individuals with OPC.

## Materials and Methods

**Plant materials**

The different organs of some medicinal plants identified by Iranian Forest Organization were selected as follows: *My. communis* leaves (Kerman province, voucher no. 1357), *Z. officinale roscoe* rhizomes (Shiraz province, voucher no. 24999), *Ma. chamomilla* flowers (Yasuj province, voucher no. 1387-1), *T. ammi* seeds (Isfahan province, voucher no. 15125) and *O. vulgare* leaves (Kurdistan province, voucher no. 9428).

**Antifungal drugs**

Fluconazole (FLU) powder (Janssen Pharmaceutica, Beerse, Belgium) was purchased, dissolved in sterile distilled water and stored as 0.25 ml aliquots at -20^o^C.

**Extraction of essential oils**

 The plant powders (100 g) were subjected to hydrodistillation using a Clevenger-type apparatus for 3 h. The collected essential oils were dried with anhydrous sodium sulphate, filtered and stored at 4-6^o^C in the dark (Council of Europe, 1997 ▶). 

**GC-MS analysis of essential oils**

GC-MS analyses were carried out in a Hewlett Packard 6890 gas chromatograph fitted with a HP1 fused silica column (polydimethylsiloxane, × 0.25 mm i.d., film thickness 0.25 mm), interfaced with a Hewlett Packard mass selective detector 5973 (Agilent Technologies) operated by HP Enhanced ChemStation software, version A.03.00. GC parameters were as above, while the other parameters were: interface temperature 250^o^C; MS source temperature 230^o^C; MS quadrupole temperature 150^o^C; ionization energy 70 eV; ionization current 60 mA; scan range 35-350 u; scans s^_^1 4.51. The identity of the compounds was achieved from their retention indices on both SPB-1 and SupelcoWax-10 columns and from their mass spectra. Retention indices, calculated by linear interpolation relative to retention times of C8-C22 n-alkanes, were compared with those of authentic samples included in our own laboratory database. The acquired mass spectra were compared with corresponding data of components of reference oils and commercially available standards from a home-made library or from literature data (Adams, 2004 ▶). 

***C. albicans*** **isolates and inoculum preparation**

 A total of 60 *C. albicans* isolates (30 FLU-resistant and 30 FLU-susceptible strains) were obtained from HIV positive patients with OPC. All subjects gave informed consent to participate in the study. In order to identify the yeasts, germ tube test, CHROM agar, β-glucosidase test, urease test, cornmeal agar-Tween 80, sugar fermentation and assimilation tests by RAPID yeast plus system (Remel Inc., USA) were carried out. The organisms were cultured in Sabouraud dextrose agar (Difco, Spark, MD, USA) for 48 h at 35^o^C. The count of yeasts was adjusted to yield 1.5×10^8^ CFU.ml-1 using the standard McFarland counting method.

**Screening of essential oils for anti-** ***C. albicans*** **activity**

*C. albicans* isolates were classified based on FLU susceptibility according to CLSI guideline (M44-A) (NCCLS, 2004). FLU break points were considered susceptible (≥ 19 mm) and resistant (≤ 14 mm) for disc diffusion method. Minimal inhibitory concentration (MIC) and minimum fungicidal concentration (MFC) were determined according to the reference documents M27-A2 for yeasts with modifications (CLSI, 2002). In brief, stock solutions of the essential oils (10 mg.ml-1) were prepared in sterile Sabouraud dextrose broth. Serial dilutions of the stock solution of each essential oil were prepared as follows: *T. ammi* (200 to 1000 μg.ml-1), *My. communis* (1000 to 5000 μg.ml-1), *Z. officinale roscoe* (1000 to 5000 μg.ml-1), *Ma. chamomilla* (1000 to 3000 μg.ml-1) and *O. vulgare* (100 to 500 μg.ml-1) in glass tubes. Fifty microliters of the yeast suspension were added to each tube containing 100 μl of the oil and 900 μl of Sabouraud dextrose broth, and all tubes were incubated at 35^o^C for 48 h. Tubes containing only the Sabouraud dextrose broth, but without microorganisms, were used as controls. The lowest oil concentrations inhibiting fungal growth were identified as MICs. To determine MFCs, a loopful of broth was removed from each individual tube and spot-inoculated on individual Sabouraud dextrose agar plates. The plates were incubated at 35^o^C for 48 h, and MFCs were defined as the corresponding concentrations required to kill 99.9% of the cells.

**Statistical analysis**

The antifungal activity was analyzed by the two-tailed paired student’s t-test. Statistical significance was considered a *P* value less than 0.05.

## Results

The hydrodistillation of the aerial parts of the above-mentioned plants gave yellowish essential oils and their components were illustrated in Table 1. As a results of GC-MS analyses, the main components were found to be linalool (42%), thymol (25.10%) and α-terpineol (10%) in *O. vulgare*, α-pinene (27.87%), 1,8-cineole (20.15%) and linalool (10.26%) in *My. communis*, α-pinene (22.10%), camphene (10.10%) and bisabolol oxide (8.45%) in *Ma. chamomilla*, thymol (63.40%), ρ-cymene (19%) and γ-terpinene (16.90%) in *T. ammi* and zingiberene (31.79%), AR-curcumene (15.88%) and β -sesquiphellandrene (15.57%) in *Z. officinale roscoe.*

 In this study, the MIC and MFC values of the active oils were established for *C. albicans* and the results were shown in Table 2. The MIC values demonstrated the existence of inhibitory effects on *C. albicans *tested, with MIC values ranging from 300 to 3200 μg.ml-1for FLU-resistant strains and 300 to 3000 μg.ml-1for FLU-susceptible strains. Among the plants tested, the most activity was observed with *O. vulgare* and *T. ammi* essential oils showing the lower MIC values against *C. albicans* (P< 0.05), followed by *Ma. chamomilla*, *Z. officinale* *roscoe* and *My. communis* oils for understudy groups.

## Discussion

The antimicrobial properties of volatile aromatic oils from plants have been recognized since antiquity. Here, we evaluated the antifungal activities of essential oils obtained from plants against both FLU-susceptible and -resistant *C*. *albicans* strains (Shokri et al., 2012). Our findings showed that *O. vulgare* essential oil contained linalool (42%), thymol (25.10%) and α-terpineol (10%) as the main components. The essential oil of *O. vulgare* was analyzed by several authors. The main components were reported as thymol, α -terpineol, linalyl acetate and linalool. In this study, the GC-MS analysis showed that the essential oil of *T. ammi* was rich in thymol (63.40%), ρ-cymene (19%) and γ-terpinene (16.90%), and the compositions were similar to the oil compositions of previous investigations (Pérez et al., 2007; Martino et al., 2009 ▶). In overall, it seems that thymol was active against various fungal pathogens, in particular *C. albicans* (Abbaszadeh et al., 2014 ▶). 

**Table 1:** The compositions of some Iranian essential oils identified by gas chromatography/mass spectroscopy (GC-MS

***Origanum vulgare***			***Trachyspermum ammi***			***Zingiber officinale roscoe***			***Myrtus communis***			***Matricaria chamomilla***		**No.**		
42%	Linalool	63.40%		Thymol	31.79%		Zingiberene	27.87%		α-Pinene	22.10%		α-Pinene		**1**
25.10%	Thymol	19%		ρ-Cymene	15.88%		AR-Curcumene	20.15%		1,8-Cineole	10.1%		Camphene		**2**
10%	α-Terpineol	16.90%		γ-Terpinene	15.57%		β -Sesquiphellandrene	10.26%		Linalool	8.45%		Bisabolol oxide		**3**
5.70%	Z-Caryohyllene	0.40%		Sabinene	9.29%		β -Bisabolene	7.64%		α-Terpineol	6.35%		α-Bisabolol		**4**
3.40%	Borneol	0.10%		β-Ocimene	5.71%		α -Farnesene	6.17%		Linalyl acetate	5.64%		Limonene		**5**
2.30%	Germanyl acetate	0.07%		α-Thujene	3.56%		γ -Cadinene	4.87%		Germanyl acetate	4%		Sabinene		**6**
2%	1,8-Cineole	0.06%		α-Pinene	3.23%		α -Eudesmol	4.04%		α-Terpinyl acetate	4%		Camphor		**7**
1.70%	γ -Bisabolene	-		-	2.35%		4, 5-Dimethy1-11-Methylene Tricycle	1.57%		Caryophyllene oxide	3.45%		1,8-Cineole		**8**
1.60%	Camphor	-		-	2.01%		Nerolidol	1.57%		trans-Caryophyllene	1.60%		α-Terpinene		**9**
1.50%	Bicyclogermacrene	-		-	2%		7- α -(1-Hydroxyl-1-Methylethyl)	1.48%		Methyl eugenol	1%		γ-Terpinene		**10**
1.30%	γ-Terpinene	-		-	1.37%		Geraly acetate	1.35%		α-Humulene	0.90%		Germacrene D		**11**
1%	Terpinen-4-ol	-		-	1.10%		Germacrene B	0.88%		β -Pinene	0.89%		E-Sabinol		**12**
0.50%	ρ-Cymene	-		-	0.97%		Geraniol	0.67%		4-Terpineol	0.87%		Z-Sabinenehydrate		**13**
0.30%	α-Pinene	-		-	0.97%		Endo- Borneol	0.63%		δ-3-Carene	0.85%		Bicyclogermacrene		**14**
0.30%	Camphene	-		-	0.93%		β -Phellandrene	0.59%		γ-Terpinene	0.76%		Spathulenol		**15**
-	-	-		-	0.73%		Camphene	0.54%		α-Thujene	0.74%		4-Terpineol		**16**
-	-	-		-	0.64%		δ -Cadinene	1.93%		Others	0.68%		E-β-farnesene		**17**
-	-	-		-	-		-	-		-	0.69%		α-Terpineol		**18**
-	-	-		-	-		-	-		-	0.65%		Borneol		**19**
-	-	-		-	-		-	-		-	0.65%		α-Funebrene		**20**
											5.12		Others		**21**
**9** **8.7** **%**	**-**	**99.9%**		**-**	**97.1%**		**-**	**92.21%**		-	**8** **0** **.** **76** **%**		-		**Total**

**Table 2:** Minimal inhibitory concentration (MIC) and minimum fungicidal concentration (MFC) of some essential oils against *Candida albicans* isolates from HIV positive patients with oropharyngeal candidiasis

**Antifungal agents**	**FLU-resistant (Mean±SD)**		** FLU-susceptible (Mean±SD)**	
	**MIC (μg.ml** ^-1^ **)**	**MFC (μg.ml** ^-1^ **)**	**MIC (μg.ml** ^-1^ **)**	**MFC (μg.ml** ^-1^ **)**
***Trachyspermum ammi***	320±44.72	420±44.72	320±44.72	400±70.71
***Myrtus communis***	3200±447.21	3800±570.08	3000±0	3600±223.60
***Zingiber officinale roscoe***	2500±0	3000±0	2500±0	3100±223.60
***Matricaria chamomilla***	1700±410.79	2300±273.86	1550±410.79	2200±273.86
***Origanum vulgare***	300±0	400±0	300±0	400±0

As a result of GC–MS analyses, α-pinene (27.87%), α-pinene (22.10%) and zingiberene (31.79%) were as the major compounds in *My. communis*, *Ma. chamomilla *and *Z. officinale roscoe* oils, respectively. These findings are in agreement with previous works carried out by other investigators (Sultan et al., 2005 ▶; Ayoughi et al., 2011 ▶; Nabavizadeh et al., 2014 ▶). Minor differences in different studies can be due to physiological variation, genetic factors and the evolution as well as the harvest time and period.

 The present study demonstrated the inhibitory activity of essential oils ranging from 300 to 3200 μg.ml-1 for FLU-resistant and 300 to 3000 μg.ml-1 for FLU-susceptible *C. albicans* strains. Our results confirmed that *My. communis, Z. officinale roscoe, Ma. Chamomilla, T. ammi* and *O. vulgare* essential oils have antifungal activities against both FLU-susceptible and -resistant *C. albicans* isolates. The inhibitory effect of the essential oils increased when the concentration of essential oil was changed. These results have been reported in other studies as well (Motsei et al., 2003 ▶). Our results revealed the best inhibitory effect on *C. albicans* growth by *O. vulgare* and *T. ammi* oils (P< 0.05). The oils of Iranian *O. vulgare* and *T. ammi* are rich in monoterpene phenols, especially thymol, indicating the most active component against fungi (Salehi Surmaghi, 2006 ▶). The antifungal mechanism of thymol is not well understood although membrane and cell wall disruption with morphological deformation, collapse and deterioration of the yeasts have been hypothesized (Ahmad et al., 2011 ▶).

When FLU-susceptible and -resistant *C. albicans* had their susceptibility to essential oils, compared with each other, the FLU-susceptible *C. albicans* group was more susceptible than FLU-resistant one. Although the FLU-susceptible group was more sensitive to treatment with essential oils, there weren’t any significant differences between them. The comparison of susceptibility profiles showed that the FLU-resistant group was as susceptible as FLU-susceptible group. This fact suggests that the mechanism of antifungal action of essential oils is independent of changes associated with resistance to azoles (Pozzatti et al., 2008 ▶). Such an interesting result warrants further studies including other FLU-resistant fungi. Regarding chemical composition, *T. ammi* and *O. vulgare* essential oils showed thymol at concentrations of 63.4% and 25.1%, respectively. Thus, it could be the main compound responsible for its potent anti-*Candida* activity. As far as we know, this is the first study showing the activity of *T. ammi *and *O. vulgare* essential oils against FLU-resistant *C. albicans* isolates. 

The results of the susceptibility test on the *Candida* isolates indicated that the essential oils of *My. communis, Ma. chamomilla* and *Z. officinale roscoe* were less active than those obtained from *O. vulgare* and *T. ammi*. Therefore, we would suggest that although the thymol content was found to be higher in *O. vulgare* and *T. ammi*, the absence of it probably determined reduced antifungal activity. The comparison between groups of *C. albicans* isolates indicated that FLU-resistant *C. albicans* strains were more susceptible to *My. communis* essential oil than FLU-susceptible isolates. Similar results were observed for *Ma. chamomilla* and *Z. officinale roscoe*. Is it possible that the presence of compounds such as α-pinene or zingiberene in the three most efficient essential oils mentioned above is responsible for the activity against FLU-resistant isolates and biochemical and physiological changes secondary to development of resistance make the isolates more susceptible to some compounds? Accurate answers to these questions would require new and more thorough assays and could be the key for the development of innovative molecules with antifungal activity. Our results are in agreement with Pozzatti et al. (2008) ▶ and Vazquez et al. (2005) ▶ studies.

 In conclusion, *O. vulgare* and *T. ammi* essential oils showed high antifungal activity when compared with those of *My. communis, Ma. chamomilla* and *Z. officinale roscoe *oils. In this study, differences on susceptibility between groups of *C. albicans* were observed; indicating that the FLU-resistant group was more susceptible to these essential oils than the FLU-susceptible group.
